# The 2024 Spain Floods: A Call for Resilience and the Duty of Memory

**DOI:** 10.3389/ijph.2025.1608236

**Published:** 2025-02-12

**Authors:** Periklis Charalampous, Niko Speybroeck, Joris A. F. van Loenhout, Gurvan Pluen, Damien Delforge

**Affiliations:** ^1^ Institute of Health and Society (IRSS), Université catholique de Louvain, Brussels, Belgium; ^2^ Department of Public Health, Erasmus Medical Center, Rotterdam, Netherlands; ^3^ Department of Epidemiology and Public Health, Sciensano, Brussels, Belgium

**Keywords:** disaster preparedness, floods, emergency health, climate change, Spain, disaster risk reduction, disaster loss database, Emergency Events Database (EM-DAT)

**FIGURE 1:**
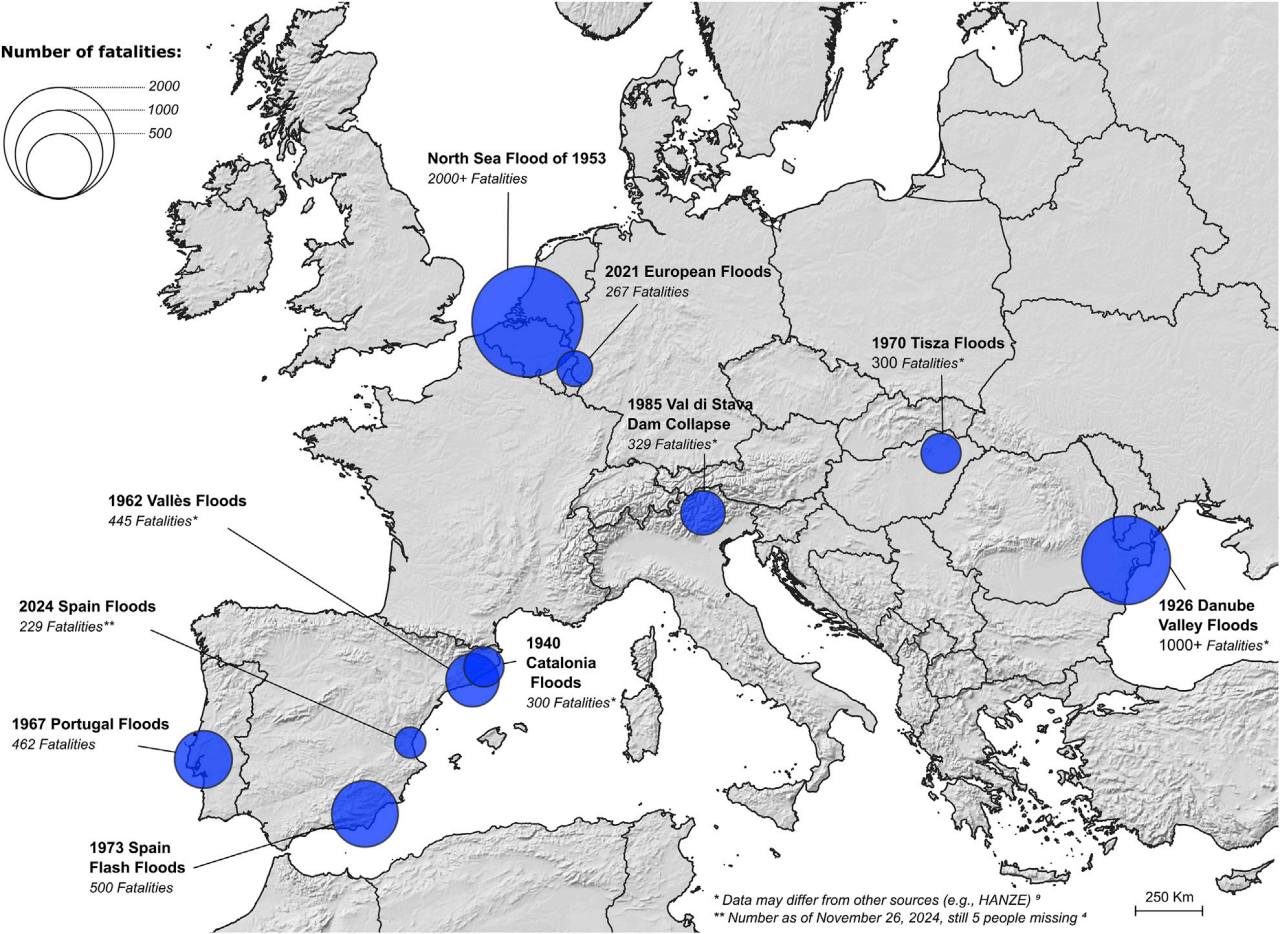
Top-10 deadliest flood events in Europe, 1900–2024, from the Emergency Events Database (EM-DAT) [3].

Floods have become increasingly frequent globally, driven by rising sea levels, urbanisation, changes in land use and extreme precipitation [1]. Over the past three decades, floods have caused over 218,000 fatalities, with a third occurring in South-East Asia [2, 3]. Europe has witnessed various severe flooding events, such as the 1926 Danube Valley Floods in Romania, with likely more than 1,000 fatalities; the 1953 North Sea Flood, which inundated the Netherlands, the UK, Belgium, and Luxembourg, resulting in over 2,000 fatalities; and the 1973 Spain Flash Floods, which caused 500 fatalities (Figure 1) [3].

Most recently, the October-November 2024 Valencia floods in Spain are reported to have caused over 220 deaths, displaced thousands [4], and are considered the deadliest Spanish flood since 1973 (Figure 1). These floods were triggered by *gota fría* (or “cold drop”), a phenomenon where cold air meets warm Mediterranean waters, leading to intense rainfall.

Flood disasters stem from complex interactions of environmental and human factors. In Spain, as in much of Europe, floods often coincide with other hazards like storms, landslides, or infrastructure failures, causing significant economic and human loss. Preventing future disasters requires understanding the interplay of exposure, vulnerability, and capacity, alongside effective risk communication throughout all stages of disaster management, from emergency response to public awareness. Advancements over recent decades, such as the United Nations’ 2015–2030 Sendai Framework for Disaster Risk Reduction [5], the European Union’s 2007 Flood Directive [6], and tools like the European Flood Awareness System [7] have improved preparedness and early warning capabilities.

However, flood risks continue to grow, necessitating stronger evidence-based practices. Databases such as the Emergency Events Database [3, 8] and HANZE [9] are invaluable for analysing past events and identifying links between human, economic and environmental factors.

The 2024 Spain Flood is stark reminder of the need to nurture a culture of proactive risk management, a commitment already established 35 years ago during the 1990 International Decade for Natural Disaster Reduction [10]. While tragic, disasters can inspire cultural and governmental dedication to prevention through preparation, coordination and documentation. In embracing resilience through remembrance, we fulfill our duty to honour those affected—both the deceased and the living—with compassion and commitment to a safer future.
